# Alcohol consumption and its associated factors among pregnant women in Sub-Saharan Africa: a systematic review and meta-analysis' as given in the submission system

**DOI:** 10.1186/s13011-020-00269-3

**Published:** 2020-04-15

**Authors:** Alemu Earsido Addila, Telake Azale Bisetegn, Yigzaw Kebede Gete, Mezgebu Yitayal Mengistu, Getnet Mihretie Beyene

**Affiliations:** 1 Department of Public Health, College of Medicine and Health Sciences, Wachemo University, Hossana, Ethiopia; 2 Department of Epidemiology and Biostatistics, Institute of Public Health, College of Medicine and Health Sciences, University of Gondar, Gondar, Ethiopia; 3 Department of Health Promotion and Behavioral Sciences, Institute of Public Health, College of Medicine and Health Sciences, University of Gondar, Gondar, Ethiopia; 4 Department of Health Systems and Policy, Institute of Public Health, College of medicine and health Sciences, University of Gondar, Gondar, Ethiopia; 5 Department of Psychiatry, College of Medicine and Health sciences, Debre Tabor University, Debra Tabor, Ethiopia

**Keywords:** Pregnant women, Alcohol consumption, Sub-Saharan Africa

## Abstract

**Background:**

Alcohol consumption during pregnancy represents a significant public health concern. It has several adverse health effects for both the mother and the developing fetus. This study aimed to estimate the pooled prevalence and the effect size of associated factors of alcohol consumption during pregnancy in Sub-Saharan Africa countries.

**Methods:**

The results of the review were reported based on the Preferred Reporting Items for Systematic Review and Meta-Analysis statement (PRISMA) guideline and, it was registered in the Prospero database, number CRD42019127103. The available primary studies were collated from different databases: PubMed, CINAHL, Cochrane Library, PsycINFO, Google Scholar, African Journals Online and Centre for Addiction and Mental Health Library. The main search terms were [((alcohol consumption) OR (alcohol drinking) OR (alcohol use) OR (ethanol use) OR (alcohol exposure)) AND ((pregnant women) OR (pregnant mother) OR (during pregnancy)) AND (Sub-Saharan Africa)]. We used the Joanna Briggs Institute (JBI) for critical appraisal of studies. The random-effects model was computed to estimate the pooled prevalence. Heterogeneity between studies was checked using the I^2^ statistic and the Cochrane Q test.

**Results:**

The review resulted in 963 original studies after searching various databases, and finally 37 studies in qualitative synthesis and 30 articles in the systematic review and meta-analysis were included. The overall summary estimate of the prevalence of alcohol consumption during pregnancy was found to be 20.83% (95% CI: 18.21, 23.46). The pooled estimate of meta-analysis showed that depression (OR: 1.572; 95% CI: 1.34, 1.845), partners’ alcohol use (OR: 1.32, 95% CI: 1.11, 1.57), knowledge on harmful effect of alcohol consumption (OR: 0.36, 95% CI: 0.29, 0.45) and, unplanned pregnancy (OR: 2.33, 95% CI: 1.17, 4.63) were statistically significant factors with alcohol consumption during pregnancy.

**Conclusions:**

The result showed that there was high alcohol consumption during pregnancy in Sub- Saharan Africa. Alcohol consumption during pregnancy was associated with depression, partners’ alcohol use, unplanned pregnancy and knowledge of the harmful effects of alcohol consumption. Therefore, this will be a basis for public policy and resource allocation for prevention initiatives.

## Background

Maternal mortality is still a major public health challenge. Most of these deaths are preventable and related to modifiable risk behaviors. Alcohol drinking during pregnancy is one of the few preventable and adjustable risk factors for adverse pregnancy and birth outcomes [1, 2].

Alcohol drinking during pregnancy represents a significant public health concern. It has several adverse health effects for both the mother and the developing fetus [3]. Because of this, there is no universally accepted safe amount and time of alcohol consumption during pregnancy. Almost all guidelines advocate for women who are pregnant or who want to conceive to abstain from any amount of alcohol consumption because it is a known teratogen and causes potential adverse effects on pregnancy and birth outcomes [4–9]. Alcohol consumption during pregnancy can cause miscarriage, stillbirth, premature birth, congenital malformations, intrauterine growth retardation, and low birth weight. It is also attributed to fetal alcohol spectrum disorders (FASDs), (a group of conditions related to alcohol exposure before birth characterized by a range of lifelong irreversible negative health impacts, like physical, behavioral, and intellectual disabilities); and a leading cause of non-genetic mental retardation and other neurodevelopment deficits [5, 8–11]. Some studies on the relationship between these risks and the amount of alcohol consumed suggest that low-to-moderate drinking does not result in the same outcomes as heavy drinking [10, 12]. On the other hand, new findings advocate that a small amount of alcohol consumption during the second and third trimester is not harmful to the unborn baby [10, 13]. Generally, alcohol consumption during pregnancy is a common risk factor for the health of newborns and the mother.

Among many countries in the world, women who are living in Chad, Namibia, Uganda, and Ethiopia are the highest alcohol consumers which are ranging from 17.7 to 24.5 l of pure alcohol per capita per year [14–16]. Different studies conducted in various settings in Africa countries reveal that the prevalence of alcohol consumption during pregnancy varies from 2.5% [17] to 59.28% [18] which shows that a great discrepancy across different geographical settings and at different periods. In Sub-Saharan Africa, a great number of unplanned pregnancy [19], lack of awareness about the effect of alcohol consumption [20], having partners and friends consume alcohol [19, 21], some health-related problems like depression [22] and unemployment [19] are the main factors for alcohol consumption during pregnancy. The results showed that questionable reports on the magnitude of alcohol consumption and its associated factors during pregnancy. Moreover, there is no recent representative pooled data on alcohol consumption during pregnancy in Sub-Saharan Africa. Systematic reviews and meta-analysis were previous computed in the WHO African Region, however, things are dynamic and updated information is very essential for policy-makers and program-implementers. In order to take appropriate action, estimating the pooled magnitude and identifying factors influencing alcohol consumption are very crucial. Therefore, this systematic review and meta-analysis aimed to estimate the pooled prevalence and the effect size of associated factors of alcohol consumption during pregnancy in Sub-Saharan Africa countries.

## Methods

Preferred Reporting Items for Systematic Review and Meta-Analysis statement (PRISMA) flow diagram and checklist were applied for designing and reporting the procedure [23] and, it was registered in the Prospero database, number CRD42019127103 (available at https://www.crd.york.ac.uk/PROSPERO/#myprospero).

### Eligibility criteria

Cross-sectional, case-control, and cohort studies were included in the review. Studies that had reported the prevalence and/or at least one associated factor of alcohol consumption during pregnancy and were published in the English language from November 2009 to January 30, 2020, considered for review. Any amount of alcohol drinking during pregnancy and its risk factors were considered. Articles without abstracts and/or full text, and where there was the difficulty of extracting data were excluded from the analysis.

### Searching strategy and data sources

Primary studies were collated from different databases (PubMed, CINAHL, Cochrane Library, PsycINFO, Google Scholar, Global Index Medicus, EMBASE, African Journals Online (AJOL) and Centre for Addiction and Mental Health Library).

The following search key terms were used by combing: “alcohol consumption”,” alcohol drinking”, alcohol use”, pregnant women”,” pregnant mother”, “prenatal”, “prevalence”, “frequency”, “occurrence”, “epidemiology”, “determinant factors”, “associated factors”, “influencing factors”,” predictors”, “contributing factors” and “Sub-Saharan Africa”(all Sub-Saharan Africa countries were entered turn by turn). Using all those terms, the search strategies were developed using different Boolean operators like ‘AND’ and ‘OR’ and MeSH Terms accordingly. The main search terms were [((alcohol consumption) OR (alcohol drinking) OR (alcohol use) OR (ethanol use) OR (alcohol exposure)) AND ((pregnant women) OR (pregnant mother) OR (during pregnancy)) AND (Sub-Saharan Africa)] from PubMed. The search was conducted turn by turn in all (46 according to UN Development Program) Sub-Saharan Africa countries [24] besides the term “Sub-Saharan Africa“.

### Study selection

Two independent reviewers (A.E and G.M) screened titles, abstracts and full texts for inclusion which were exported through data manager Endnote 7 to identify relevant articles and to remove unnecessary duplications. The divergences between two reviewers (A.E and G.M) were managed by a discussion based on established criteria. The remaining articles were evaluated based on the title, language and study setting. After rejecting inappropriate titles, the relevant abstracts and full texts were thoroughly reviewed.

### Quality assessment

The Joanna Briggs Institute (JBI) critical appraisal checklist was used to evaluate the quality of articles [25–27]. The checklist comprises of 8, 10, and 11 items for cross-sectional, case-control and cohort studies, respectively. The assessments of both reviewers (A. E and G. M) were compared and thoroughly discussed before a decisive quality assessment was made.

### Data extraction

The eligible studies were identified using a checklist, and the data were extracted using a structured format on a Microsoft Excel spreadsheet by two independent reviewers (A.E and G.M). After the extracted data were cross-checked; some data extraction processes were repeated because of disagreement between reviewers. The Microsoft Excel spreadsheet was used to extract the most relevant information for review which comprises of the name of the first author, year of the publication, the study country, the study design, the sample size, the study setting and the instrument used to get alcohol use data.

### Outcome variable

The outcome of the study was alcohol consumption which was defined as the proportion of pregnant women who consumed any amount of alcohol during pregnancy.

### Statistical analysis

We used a random-effects model to estimate the pooled prevalence of alcohol consumption during pregnancy. Because we assumed that the parameter of interest was not identical across studies and the variability between the observed proportions. Heterogeneity between studies was computed using the I^2^ statistic (amount of heterogeneity) and the Cochrane Q test (presence of heterogeneity). I^2^ values of 25, 50, and 75% were low, moderate, and high heterogeneity, respectively [28]. Sensitivity analysis was done to identify the effect of an influential study on the overall results of the study. Subgroup analysis was also conducted by study setting and country to see their contribution as the source of heterogeneity. Publication bias was assessed using Funnel plot asymmetry through visual inspection and Egger’s test [29]. Data were analyzed using statistical software Stata version 14.0.

## Results

**Fig. 1:**
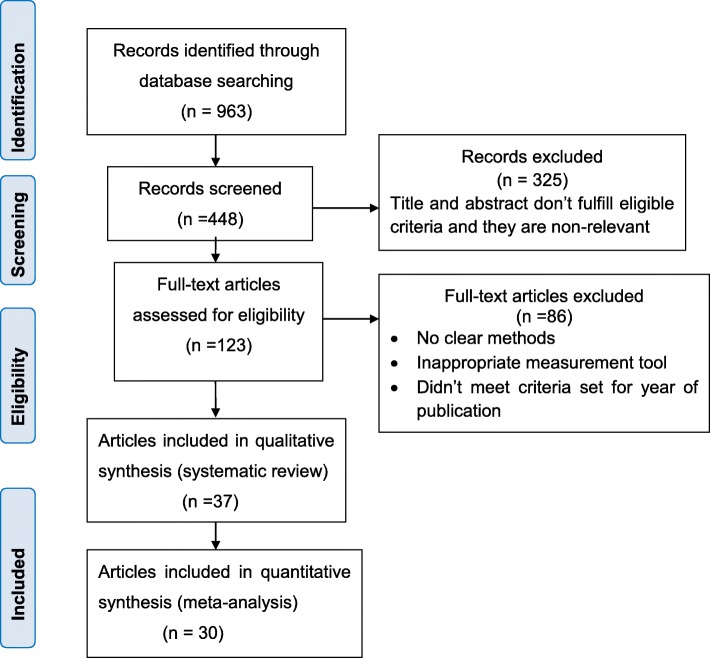
Flow diagram showing the procedure of selecting studies on alcohol consumption during pregnancy for systematic review and meta-analysis, November 2009–January 2020, Sub-Saharan Africa

A total of 963 articles were retrieved after a comprehensive search strategy from different databases. Majority of articles 384 (39.9%) were found in PubMed (after duplicated articles were specifically taken out from the PubMed database) and the rest from other databases. In the process, 448 records remained after removing duplicated retrievals and of which 325 records were excluded after assessing titles and abstracts because they were found to be irrelevant and did not fulfill the eligibility criteria. Thorough assessment and screening of full texts were done for the remaining 123 articles. Of these, 86 records were rejected for the absence of clear methods, inappropriate measurement tools and unmet criteria set for the year of publication. Thirty-seven studies were included in the qualitative synthesis. Finally, 30 studies were eligible for systematic review and meta-analysis (Fig.1).

In the final review, articles were published from 10 countries among 46 countries were included. Of these studies, eleven studies were found from Ethiopia [13, 19, 30–38], nine articles were from South Africa [20, 22, 39–46], five articles were from Nigeria [18, 47–50], two studies were from Ghana [51, 52], one article was from Kenya [53], three articles were from Tanzania [54–56], two studies were from Uganda [21, 57] one study was from Burkina Faso [58], and one article was from Zambia [59]. Regarding the study designs, twenty-five (83%) of them were cross-sectional, three studies (3.5%) were cohort and three articles (3.5%) were case-control. In all studies, alcohol consumption data were taken from the self-report of pregnant women, which means no data was collected using biomarkers or meconium tests. Lists of self-reporting questionnaires were: Alcohol Use Disorder Identification Test-Consumption (AUDIT-C) [22, 40, 55], Tolerance, Worried, Eye-opener, Amnesia and, K/Cut-down (TWEAK) [57], Alcohol Use Disorders Identification Test (AUDIT) [39, 41], Cutting down, Annoyance, Guilt, and Eye-opener (CAGE) [21], Alcohol Smoking and Substance Involvement Test (ASSIST) [44, 50], Tolerance- Annoyed, Cut off, Eye-opening (T-ACE) [19], and 1-Question screen strategy [60]. Structured questionnaires were used in the rest of all studies (64%) to screen alcohol consumption during pregnancy.

### Prevalence of alcohol consumption during pregnancy of primary studies

A total of 30 studies with 17,908 participants were used for prevalence estimation. The review indicated that the prevalence of alcohol consumption among pregnant women varied widely across countries. The lowest prevalence (4.3%) was found in a study carried out at Dessie referral hospital, Northeast Ethiopia [34], whereas the highest prevalence (59.28%) was reported in research conducted at a tertiary hospital in South Nigeria [18]. The detailed description of the included articles had been shown in Table 1.

**Table 1:** Characteristics of original studies included in systematic review and meta-analysis of alcohol consumption during pregnancy in Sub-Saharan Africa (*n* = 37)

s. no	Authors	Study country	Publication Year	Study design	Study setting	Sample size	Prevalence No (%)	Data collection tool
1.	English LL [57]	Uganda	2015	Cohort	Community based	505	81(16)	TWEAK questionnaire
2.	Namagembe, I [21].	Uganda	2010	Cross sectional	Health facility based	610	151(25)	CAGE questionnaire
3.	Mpelo, Matunga [55]	Tanzania	2018	Case-control	Health facility based	365	55(15.1)	AUDIT-C questionnaire
4.	Mosha, T. C [54].	Tanzania	2010	Cohort	Health facility based	157	10(6.4)	Structured questionnaire
5.	Nombo, A [56].	Tanzania	2018	Cross sectional	Health facility based	609	77(12.6)	Standardized questionnaire
6.	Hartley, M [22].	South Africa	2011	Cross sectional	Health facility based	1062	295(27.8)	AUDIT-C questionnaire
7.	Myers, B [44]	South Africa	2017	Cross sectional	Health facility based	667	148(22)	ASSIST questionnaire
8.	Bundree, S [43].	South Africa	2017	Cohort	Community based	759	139(18)	structured questionnaire
9.	Morojele, N [20].	South Africa	2010	Cross sectional	Community based	1018	292(28.7)	structured questionnaire
10.	Vythilingum, B [39].	South Africa	2012	Cross sectional	Health facility based	323	66(20.2)	AUDIT questionnaire
11.	Tomlinson, M [40].	South Africa	2014	Cross sectional	Health facility based	1145	284(25)	AUDIT-C, structured questionnaire
12.	Onah, M [42].	South Africa	2016	Cross sectional	Health facility based	376	52(13.8)	structured questionnaire
13.	Roos, A [41].	South Africa	2015	Cross sectional	Health facility based	148	43(29)	AUDIT questionnaire
14.	O’Connor, M.J [46].	South Africa	2011	Cross sectional	Community based	619	167(27)	AUDIT-C, structured questionnaire
15	Ordinioha, B [18].	Nigeria	2015	Cross sectional	Health facility based	221	131(59.28)	Structured questionnaire
16.	Adebowale, O [50].	Nigeria	2018	Cross sectional	Health facility based	395	67(17)	ASSIST/self-reporting questionnaire
17.	Nyoyoko, N [48].	Nigeria	2016	Cross sectional	Health facility based	502	153(30.5)	semi-structured
18.	Onwuka, C [47].	Nigeria	2016	Cross sectional	Health facility based	380	86(22.6)	structured questionnaire
19.	Thompson,O [49].	Nigeria	2016	Cross sectional	Health facility based	294	17(5.8)	structured questionnaire
20.	Wagura,P [53].	Kenya	2018	Cohort	Health facility based	322	21(6.5)	standardized questionnaire
21.	Otupiri, E [51]	Ghana	2012	Cross sectional	Health facility based	397	81(20.4)	structured questionnaire
22.	Lekettey, J [61].	Ghana	2017	Cohort	Health facility based	250	120(48)	Structured questionnaire
23.	Taye, M [33].	Ethiopia	2018	Case-control	Health facility based	414	67(19.3)	Structured questionnaire
24.	Anteab, K [19]	Ethiopia	2014	Case-control	Health facility based	810	275(34)	T-ACE, standardized questionnaire
25.	Endashew, M [31].	Ethiopia	2015	Cohort	Health facility based	453	202(44.9)	Standardized questionnaire
26.	Grum,T [13].	Ethiopia	2017	Cross sectional	Health facility based	291	54(18.9)	structured questionnaire
27.	Aboye, W [32].	Ethiopia	2018	Cross sectional	Health facility based	308	50(16.2)	structured questionnaire
28.	Demelash,H [30].	Ethiopia	2015	Cross sectional	Health facility based	387	71(18.3)	Standardized questionnaire
29.	Tessema [34]	Ethiopia	2015	Cross sectional	Health facility based	490	21(4.3)	structured questionnaire
30.	Hanlon [36]	Ethiopia	2009	Cohort	Community based	1040	52(5)	Self-Reporting Questionnaire
31.	Terefa, T [35].	Ethiopia	2015	Cross sectional	Community based	340	86(25.3)	A semi-structured,
32.	Barthélémy, [62]	DR Congo	2011	Cross sectional	Community based	240	78(32.5)	structured questionnaire
33.	Williams, A [60].	Congo	2013	Cross sectional	Health facility based	3099	722(23.3)	1-Question screen
34.	Moise, I. K.et al. [59]	Zambia	2019	Cross sectional	Health facility based	188	40(21.2)	T-ACE, standardized questionnaire
35.	Sanou, et al. [58]	Burkina Faso	2017	Cross sectional	Community based	518	96(18.5)	Self-Reporting Questionnaire (yes/no)
36.	Wubetu. A et al. [38]	Ethiopia	2019	Cross sectional	Health facility based	380	62(16.1)	CAGE questionnaire
37.	Mekuriaw B.et al. [37]	Ethiopia	2019	Cross sectional	Health facility based	759	58(8.1)	AUDIT-C questionnaire

### Associated factors explanation of original studies

The studies indicated that alcohol consumption during pregnancy associated with three major themes in Sub-Saharan Africa: socioeconomic factors like educational status, knowledge on harmful effects of alcohol use, source of income, and employment status; obstetric factors such as complications during previous pregnancies, marital status, pregnancy plan, and parity; and behavioral factors like prior alcohol consumption, partner’s alcohol use, peer pressure on alcohol use, and smoking.

The review showed that post-primary educational status as compared to primary education (AOR 10.64, 95% CI 1.89,19.84) [55], High School and TVET compared with illiterate women (AOR = 2.70; 95% CI: 1.25, 5.81) [19], having Knowledge on harmful effect of alcohol use (AOR = 0.37; 95% CI: 0.20, 0.70) [20], AOR = 3.26; 95% CI:1.79, 5.97) [47], making local brews as a source of income (AOR = 11.444; 95% CI: 1.01,19.86) [55], being unemployed (AOR = 3.11; 95% CI: 1.73, 5.60) [19] were predictors of alcohol consumption during pregnancy.

Regarding obstetric factors, not having complications in previous pregnancies (AOR = 4.93; 95% CI: 1.031,23.59) [55], being married (AOR = 3.09; 95% CI: 1.79, 5.33) [19], unplanned (AOR = 3.12; 95% CI: 1.85, 5.28) [19], (OR = 1.9; 95% CI: 1.06–3.4) [55], (AOR = 2.12; 95% CI:1.2,3.73) [37], and nulliparity (OR = 1.94; 95%CI: 1.10, 3.40) [47] were factors associated with alcohol consumption during pregnancy. On the other hand, pre-pregnancy alcohol consumption (AOR = 5.19; 95% CI: 4.791,34.87) [55], (OR = 4.522) [39], (AOR = 1.94; 95% CI:1.18, 3.18) [47], (AOR = 2.17, 95% CI: 1.18, 4.00) [37], alcohol consumption encouraged by somebody or having friends who use alcohol (AOR = 3.84; 95% CI: 2.34, 6.30) [19], (AOR = 1.57; 95% CI: 1.39,6.25) [55], (AOR = 2.36; 95% CI: 1.72, 3.23) [21], partner alcohol use (AOR = 1.71; 95% CI:1.09, 2.67) [19], (AOR = 2.58; 95% CI: 1.12, 5.97) [20], (AOR = 1.56; 95% CI:1.21, 2.02) [21], (OR = 4.87; 95% CI: 2.64–8.997) [55], smoking (AOR = 2.92; 95% CI: 1.10, 7.79) [20], (AOR = 1.23; 95% CI:1.15, 1.32) [21] were determinants of alcohol use during pregnancy.

Finally, pregnant mothers who were depressed more likely to drink alcohol than counterparts (AOR = 5.1; 95% CI: 1.7,14.9) [49], (AOR = 1.6; 95% CI:1.22, 2.1) [22], (OR = 1.55) [40], (OR = 2.64) [42], and (AOR = 1.91, 95% CI = 1.01,4.50) [38].

### Meta-analysis

**Fig. 2:**
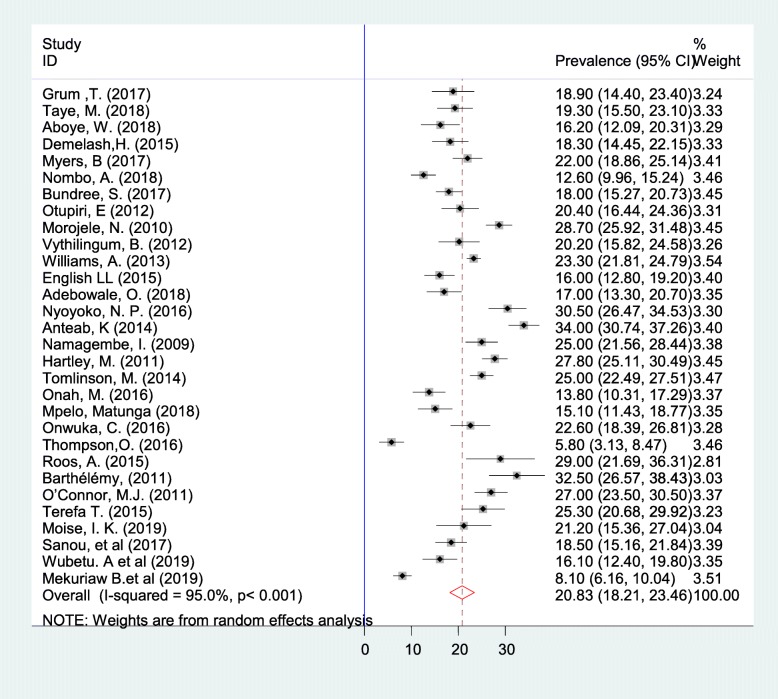
Forest plot of the prevalence of alcohol consumption during pregnancy in Sub-Saharan Africa with corresponding 95% CIs

Meta-analysis was carried out for 30 articles (17,908 participants). The overall summary estimate of the prevalence of alcohol consumption during pregnancy was found to be 20.83% (95% CI: 18.21, 23.46) by the random effect model. I-squared result (I^2^ = 95.0, *p* < 0.001) showed that there was considerable heterogeneity among the included studies (Fig.2). Since the *P*-value for the χ2 test of heterogeneity was low (< 0.05), we conducted a subgroup analysis to make out the possible sources of the observed heterogeneity.

### Subgroup analysis

**Fig. 3:**
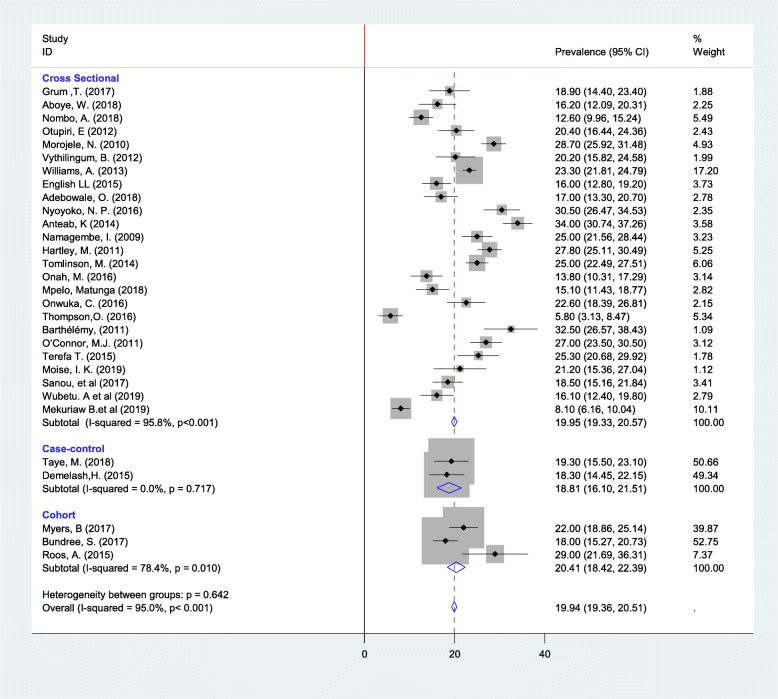
Subgroup prevalence of alcohol consumption during pregnancy in Sub-Saharan Africa with corresponding 95% CIs by study designs

We computed subgroup analysis based on study design, country and, study setting when at least two studies are in the same category. Accordingly, the lowest prevalence and heterogeneity of alcohol use during pregnancy were reported in case-control study design (Fig.3); the heterogeneity between the groups was not significant (*p* = 0.642). Concerning the study setting and country, the heterogeneity was still appreciable in both subgroups. On the other hand, the highest prevalence of alcohol consumption during pregnancy was found in DR Congo 32.50% (95%CI: 26.57, 38.43), whereas the lowest was remarked in Tanzania 13.45% (95%CI: 11.31, 15.59) (Table 2).

**Table 2:** Summary of subgroup analysis of the prevalence of alcohol consumption during pregnancy in Sub-Saharan Africa, 2009- Jan 2020 (*n* = 30)

Variables (subgroup)	Characteristics	Included studies	Pooled prevalence (95% CI)	I^2^	*P*-value
By design	Cross- sectional	25	18.81(16.10–21.50)	95%	< 0.001
	Case- control	2	18.81(16.10–21.51)	0.0%	0.717
	Cohort	3	20.41(18.42–22.39)	78.4%	0.010
By study setting	Health facility	23	17.72(17.06–18.38)	93.8%	< 0.001
	Community	7	26.77(25.61–27.93)	87.3%	0.001
By country	South Africa	9	23.54 (22.48, 24.60)	89.9%	< 0.001
	Ethiopia	8	16.79 (15.62, 17.97)	96.%	< 0.001
	Nigeria	4	15.73(13.99–17.47)	97.4%	< 0.001
	Uganda	2	20.18(11.31–15.59)	92.9%	< 0.001
	Tanzania	2	13.45(11.31–15.59)	14.9%	0.278
	Zambia	1	21.20 (15.36, 27.04)	.	.
	Burkina Faso	1	18.50 (15.16, 21.84)	.	.
	DR Congo	1	32.50 (26.57, 38.43	.	.
	Congo	1	23.30 (21.81, 24.79)	.	.
	Ghana	1	20.40 (16.44, 24.36)	.	.

### Sensitivity analysis

Sensitivity analysis was performed to identify which single study most influenced all other studies. However, an overweight study was not seen.

### Publication bias

**Fig. 4:**
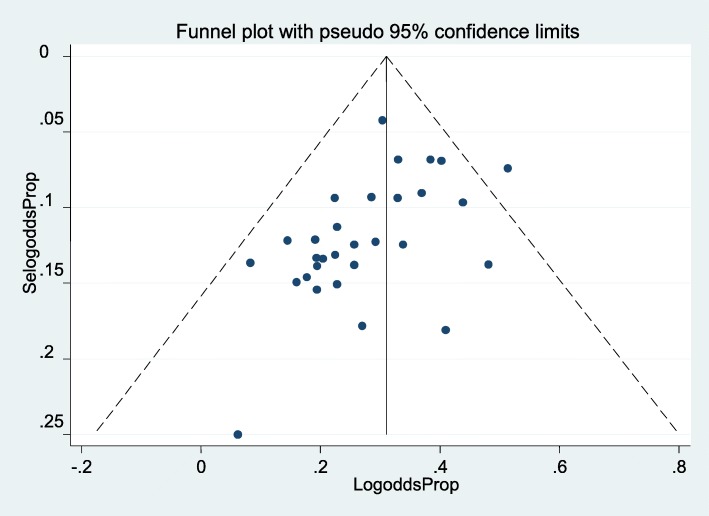
Funnel plot depicts publication bias of alcohol consumption during pregnancy for systematic review and meta-analysis in Sub-Saharan Africa

It was assessed by using a funnel plot of visual inspection which was symmetrical distribution (Fig.4) and, Egger’s regression test statistics *p*-value was 0.870, which showed the absence of publication bias for estimating the prevalence of alcohol consumption during pregnancy.

### Factors associated with alcohol consumption during pregnancy

Thirteen studies were screened which fulfilled the set criteria for factors associated with pregnant mothers alcohol use [19–22, 37–40, 42, 46, 47, 49, 63]. The result of the pooled estimate showed that depression, partners’ alcohol use, knowledge on the harmful effects of alcohol consumption and unplanned pregnancy were statistically significant factors to influence alcohol consumption during pregnancy. On the other hand, cigarette smoking, pre-pregnancy alcohol drinking and having alcohol drinking friends were not statistically significant factors for alcohol use.

### Association between pregnant mothers and their partners’ alcohol consumption

**Fig. 5:**
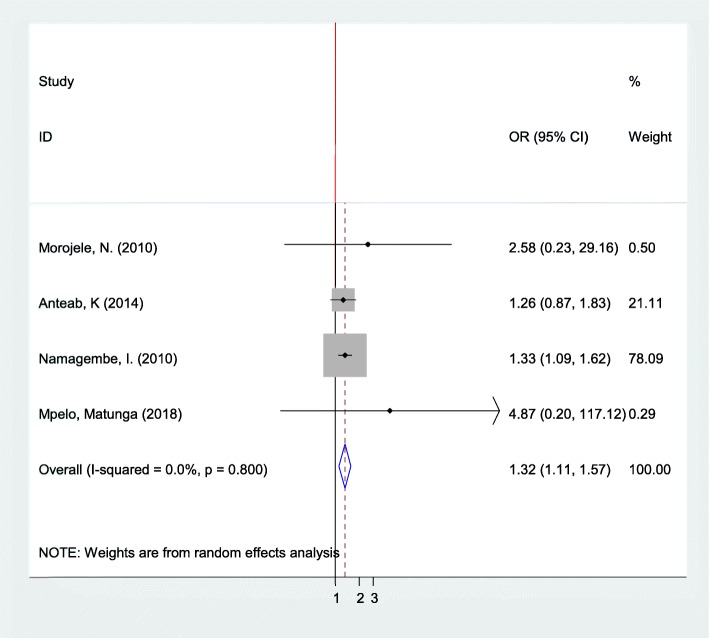
The pooled odds ratio of the association between pregnant women and their partners’ alcohol consumption in Sub-Saharan Africa

According to the pooled effects of four studies [19–21, 63], the likelihood of pregnant women alcohol consumption was 1.32 times more likely to use alcohol whose partners consumed alcohol as compared to their counterparts (OR: 1.32, 95% CI: 1.11, 1.57) (Fig. 5).

**Fig. 6:**
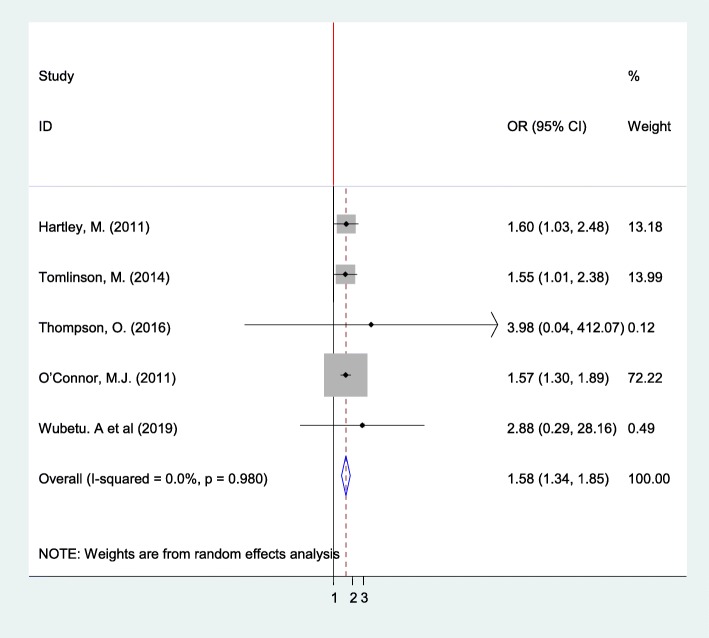
The pooled odds ratio of the association between pregnant women alcohol consumption and depression in Sub-Saharan Africa

Furthermore, publication bias was checked using Begg’s and Egger’s tests. The results of these tests showed that there was no possible presence of statistically significant publication bias (*p* = 0.308 and 0.174), respectively.

### Association between alcohol consumption of pregnant mothers and depression

The review exhibited that depression was a statistically significant factor for alcohol consumption during pregnancy based on analysis conducted on five studies [22, 38, 40, 46, 49]. Pregnant women who were depressed almost 1.6 times more likely to use alcohol than their counterparts (OR: 1.572, 95% CI: 1.34, 1.85) with I^2^ = 0.0% and *p*-value = 0.98). Publication bias was visually examined using funnel plot and tested with Egger’s weighted regression and Begg’s rank correlation method. The result of these tests showed that there was no significant asymmetry (*p* = 0.053 and 0.086), respectively (Fig. 6).

To determine the correlation between alcohol consumption of the pregnant mothers and their knowledge of the harmful effect of alcohol use on birth outcomes two studies were analyzed [20, 47]. Pregnant women who had awareness of the adverse effect of alcohol drinking during pregnancy on birth outcomes were 64% less likely to consume alcohol as compared to their counterparts (OR: 0.36, 95% CI: 0.29, 0.45) with (I^2^ = 0.0% and *p* = 0.596). Women who had unplanned their pregnancy were twice more likely to drink alcohol as compared to those who had planned [19, 37, 55] (OR: 2.33, 95% CI: 1.17, 4.63) with (I^2^ = 0.0% and *p* = 0.779). In addition, the pooled result of analysis indicated that drinking encouraged by friends or having alcohol drinking friends [19, 21, 55] (OR: 1.78, 95% CI: 0.79, 4.05) with (I^2^ = 46.8% and *p* = 0.153), pre-pregnancy alcohol consumption [37, 39, 47, 55] (OR: 2.05, 95% CI: 0.93, 4.50) with (I^2^ = 0.0% and *p* = 0.983), and cigarette smoking [20, 21, 46] (OR: 2.37, 95% CI: 0.70, 7.99) with (I^2^ = 83.3% and *p* = 0.002) were not statistically significant factors for alcohol consumption.

## Discussion

Despite the well known teratogenic adverse effect of alcohol consumption during pregnancy, several pregnant women use alcohol in Sub-Saharan Africa. Alcohol consumption during pregnancy is one of the modifiable health behaviors. Consumption of alcohol during pregnancy results in abortion-related maternal deaths and various fetal complications [1]. Pregnancy alcohol exposure (PAE) may serve as a proxy indicator of Fetal Alcohol Syndrome (FAS). To design pragmatic intervention, it is very vital to identify the women at high risk for using alcohol during pregnancy. In Sub-Saharan Africa, conducting this type of study will be paramount as input for policymakers and program planners working in the area.

We scanned 963 titles that were thoroughly specified to 37 publications for narrative synthesis and 30 studies for systematic review and meta-analysis. The search was retrieved from November 2009 to January 2020.

In this review, the prevalence of alcohol use during pregnancy varied within and across countries. For example, in Sub-Saharan Africa, the prevalence varied from 4.30 to 59.28%, while in Ethiopia; it was 4.3 to 44.9%. The variation might not be only due to maternal or individual drinking behaviors but also from environmental, genetic, political, religious, policy and cultural differences [64]. The rate of alcohol consumption during pregnancy was very high in some studies [18, 19, 31, 52] due to several reasons like socialization with friends or peer pressure, lack of adequate information about the adverse health effect of PAE and having alcohol-consuming partners. This also could be because of the weak regulatory strategy of alcohol production, promotion, drinking pattern, and availability in every grocery and bar [65]. For example, in Ethiopia, *“Areki”, “Tella” and “Tej”* are local alcoholic beverages that are produced and accessible everywhere [66]. Women’s alcohol drinking has been increasing in line with economic growth, altering gender roles and increasing the social acceptability of women’s alcohol consumption [67].

Accordingly, the pooled magnitude of alcohol consumption during the pregnancy was 20.83% (95% CI: 18.21, 23.46). This result was greater than from findings of WHO Africa region 18.52% [15], Korea 16.4% [68], global estimated prevalence 9·8% [69], and the Behavioral Risk Factor Surveillance System (BRFSS) surveys conducted by the Centers for Disease Control and Prevention in the United States the countrywide data between 2015 and 2017 11.5% [70]. On the other hand, this figure was lower than studies done in Russia 25.5–60% [71, 72], and the report of prospective cohort studies and national surveys at various times in Australian 28–72% [73–75]. The discrepancies between the drinking patterns of the pregnant mother might be associated with socioeconomic, cultural, health policy, socio-demographic, and alcohol drinkers screening tool differences. Determining the accurate prevalence and amount of alcohol consumption during pregnancy is very difficult since under-reporting is common because of social desirability bias, recall bias, religious beliefs and, seasonal and geographic variations [76, 77].

This result had indirectly shown that many pregnant women low adherence to alcohol guidelines which advising complete abstinence from alcohol use during pregnancy [4]. It might also show that there are many mothers and developing fetuses suffering from many adverse health effects in Sub-Saharan Africa associated with maternal alcohol use. A high number of pregnant women could unknowingly expose the developing fetus to alcohol.

Designing successful public health promotion policies relating to reducing alcohol consumption in pregnancy and preventing alcohol-exposed pregnancy requires an understanding of why women drink alcohol during pregnancy. This review showed that alcohol consumption during pregnancy was associated with three major themes in the original studies: socioeconomic factors like educational status, knowledge on harmful effects of alcohol use, source of income, and employment status; obstetric factors such as complications during previous pregnancies, marital status, pregnancy plan, and parity; and behavioral factors like prior alcohol consumption, partner’s alcohol use, peer pressure on alcohol use, and smoking. However, in the meta-analysis, four variables had a statistically significant association with alcohol use during pregnancy. Depression was found to be a predictor of drinking during pregnancy in five studies [22, 38, 40, 46, 49]; this finding was consistent with the systematic review at the international level [78]. The result of some studies showed that various psychiatric symptoms like depression and alcohol consumption have co-occurred in the same individual [79]. This might be attributable to some women drink alcohol as self-medication to detach from depression or depression might happen due to excessive alcohol use.

We observed a statistically significant association between partner’s alcohol use and maternal alcohol intake during pregnancy in four studies [19–21, 63]. This result was consistent with a study conducted in San Diego [80]. The possible explanation was that married or cohabiting partners tend to have concordant alcohol and other substances use patterns. Moreover, unplanned pregnancy was found to be statistically risk factors for alcohol consumption in pregnant women. It was agreed with the study carried out in Korea [68]. The possible reason for this correlation might be the social and psychological impact of unplanned pregnancy that can have the potential to force pregnant women for the initiation of alcohol or other substance use to get relief from their stress [81]. Another significant factor that related to alcohol use was knowledge. It was found that women who knew that alcohol harms the fetus were less likely to consume alcohol during pregnancy. This finding was consistent with the studies conducted in Danish [82], 85% of respondents knew that alcohol is possibly harmful to the baby and in France [83], 83% of respondents knew that alcohol in pregnancy could be harmful to the fetus and they less likely use alcohol.

Health care providers need to give compelling and brief counseling regarding strategies to avoid alcohol use in primary and prenatal care. Health care providers also should encourage drinking women to use effective contraceptive measures, therefore, it is important for reducing unplanned or unintended pregnancies. A Great effort should be done to enhance awareness of women about the adverse effect of alcohol drinking during pregnancy. The partners should be informed that the harmful effects of drinking alcohol during pregnancy and assist in achieving abstinence and support for women at risk are part of routine women’s health care. Special advice has to be given for depressed pregnant women concerning mechanisms to abstain from alcohol consumption during pregnancy.

### The strength of the study

A comprehensive search strategy and strict inclusion and exclusion criteria were used.

### The limitations of the study

The magnitude of alcohol consumption during pregnancy might be underestimated because of self-reporting which could prone to social desirability and recall bias. Since different data collection tools were used in the primary studies, the tool could not measure or determine the result of the outcome in an equal manner. The authors calculated some figures from studies that were not directly written in the results; this might be unclear for readers. Besides, reports, unpublished studies, and grey literature were not included in this review.

## Conclusions

The result showed that there was high alcohol consumption during pregnancy in Sub- Saharan Africa. Alcohol consumption during pregnancy was associated with depression, partners’ alcohol use, unplanned pregnancy and knowledge of the harmful effects of alcohol consumption. Therefore, this will be a basis for public policy and resource allocation for prevention initiatives. Antenatal care providers should also assess these factors for improved detection of women at risk for alcohol-exposed pregnancies and promote a healthy lifestyle that could be optimized. There is a need for a future study on the effect of consumption during pregnancy in Sub-Saharan Africa.

## Data Availability

All generated data during this review are included in this published article.

## Abbreviations

ASSIST: Alcohol smoking and substance involvement test
AUDIT: Alcohol use disorders identification test
AUDIT-C: Alcohol use disorder identification test-consumption
CAGE: Cutting down, annoyance, guilt, and eye-opener
FAS: Fetal alcohol syndrome
FASD: Fetal alcohol spectrum disorder
JBI: Joanna Briggs Institute
PAE: Pregnancy alcohol exposure
PRISMA: Preferred reporting items for systematic review and meta-analysis statement
T-ACE: Tolerance- annoyed, cut off, eye-opening
TWEAK: Tolerance, worried, eye-opener, amnesia and, K/Cut-down
