# Correction to: Alcohol consumption and its associated factors among pregnant women in Sub-Saharan Africa: A systematic review and meta-analysis

**DOI:** 10.1186/s13011-020-00325-y

**Published:** 2020-10-26

**Authors:** Alemu Earsido Addila, Telake Azale Bisetegn, Yigzaw Kebede Gete, Mezgebu Yitayal Mengistu, Getnet Mihretie Beyene

**Affiliations:** 1 Department of Public Health, College of Medicine and Health Sciences, Wachemo University, Hossana, Ethiopia; 2 Department of Epidemiology and Biostatistics, Institute of Public Health, College of Medicine and Health Sciences, University of Gondar, Gondar, Ethiopia; 3 Department of Health Promotion and Behavioral Sciences, Institute of Public Health, College of Medicine and Health Sciences, University of Gondar, Gondar, Ethiopia; 4 Department of Health Systems and Policy, Institute of Public Health, College of medicine and health Sciences, University of Gondar, Gondar, Ethiopia; 5 Department of Psychiatry, College of Medicine and Health sciences, Debre Tabor University, Debra Tabor, Ethiopia

**Correction to: Subst Abuse Treat Prev Policy 15, 29 (2020)**

**http://orcid.org/10.1186/s13011-020-00269-3**

Following publication of the original article [1], we have been notified that there is a mistake in the title of the article.

Currently the title is: Alcohol consumption and its associated factors among pregnant women in Sub-Saharan Africa: a systematic review and meta-analysis’ as given in the submission system. It should be changed to: Alcohol consumption and its associated factors among pregnant women in Sub-Saharan Africa: A systematic review and meta-analysis.
